# Supplementary material for: WordCommentsAnalyzer: A windows software tool for qualitative research
Source: F1000Res. 2018 Sep 4;7:536. Originally published 2018 May 3. [Version 2] doi: 10.12688/f1000research.14819.2 (PMC6137411; doi:10.12688/f1000research.14819.2)
Supplement: Supplementary file 3 [file f1000research-7-17614-s0002.tgz › b2a79496-4ada-47c4-9682-a99ce867836d.zip!/Eight Iranian Health Journals/Salmand_Iranian Journal of Ageing _15.docx]

© Salmand_Iranian Journal of Ageing . This work is licensed under CC BY-NC 4.0 (https://creativecommons.org/licenses/by-nc/4.0/).

Volume & Issue: دوره 9 - شماره 3

Title: مقایسه متغیرهای اقتصادی-اجتماعی و رضایت از زندگی سالمندان شهرستان گرگان در سال‌های 1383 و 1388

http://salmandj.uswr.ac.ir/article-1-651-fa.html

اهداف: جمعیت سالمندان و سهم آنان در جمعیت کل کشوردرحال افزایش است و به 3/7درصد در سرشماری سال 1385 رسید که این میزان نسبت به سال 1375 و در یک بازه زمانی ده ساله رشد 1/5برابری را نشان می دهد. در این مطالعه وضعیت اقتصادی، اجتماعی و رضایت آنان از زندگی در سال 1388 مورد مطالعه قرار گرفته و با نتایج به‌دست آمده در سال 1383 مقایسه گردید. مواد و روش‌ها: در یک مطالعه توصیفی تحلیلی با استفاده از یک پرسشنامه استاندارد 884 سالمند همان منطقه در سال 1388 که از طریق نمونه گیری تصادفی منظم انتخاب شدند، در حیطه مسائل اجتماعی، اقتصادی و رضایت انان از زندگی مورد بررسی و مقایسه قرار گرفت. سپس اطلاعات جمع‌آوری شده با اطلاعات مربوط به 315 سالمند ساکن مناطق تحت پوشش پایگاه‌های 4 و 5 شهر گرگان که در سال 1383 جمع آوری شده بود مورد مقایسه قرار گرفت. داده‌ها با استفاده از روش‌های آماری توصیفی و آزمون‌های آماری t مستقل و کای اسکوئر مورد تجزیه و تحلیل قرار گرفتند. یافته‌ها: سطح سواد سالمندان در سال 1388 نسبت به سال 1383 تغییر معناداری را نشان می‌داد (P=0/0002). وضعیت تاهل سالمندان نیز در این سال نسبت به دوره قبل (1383) تغییر پیدا کرده بود که این تغییر نیز از نظر آماری معنادار بود (P=0/0021). سطح اقتصادی سالمندان در سال 1388 نسبت به سال 1383 تغییر نشان می‌داد که این تغییر معنادار بود (P<0/0001). جهت گذران اوقات فراغت تمامی موارد گذراندن وقت با دوستان (P<0/001)، رفتن به پارک (P=0/0173)، رفتن به سینما (P=0/0001)، گذراندن وقت با خانواده (P=0/0435) و رفتن به مسافرت (P=0/0001) بطور معنا داری تغییر نشان می‌داد. در بخش احساس سالمندان به زندگی، متغیر پر انرژی بودن نسبت با سال 1383 تغییر معناداری نشان می‌داد (P<0/01). نتیجه‌گیری: با افزایش سن جمعیت و بزرگ‌ترشدن نسبت گروه سالمندان در جمعیت کل، مشکلات مربوط به آنان از جمله مشکلات روانی، اجتماعی و رضایت آنان از زندگی می‌باید بیشتر مورد توجه قرار گیرد. در تمام دنیا و به‌خصوص درکشور ما این مشکلات در حال افزایش و نیازمند برنامه‌ریزی جامع جهت رفع آن می‌باشد.

Title: بررسی نحوه گذران اوقات فراغت و فعالیت‌های بدنی سالمندان با تأکید بر دستگاه‌های بدن‌سازی شهرداری شهر تهران درسال 1389

http://salmandj.uswr.ac.ir/article-1-659-fa.html

اهداف: پژوهش حاضر باهدف شناخت نحوه گذران اوقات فراغت و فعالیت‌های بدنی سالمندان با تأکید بر دستگاه‌های بدن‌سازی شهرداری شهر تهران در سال 1389 صورت پذیرفت. مواد و روش‌ها: این تحقیق از نوع توصیفی بوده و 366 نفر نمونه از بین کل سالمندان (701300 نفر) به‌صورت نمونه‌گیری طبقه‌ای به‌صورت تصادفی انتخاب گردیدند. ابزار جمع آوری داده‌ها، پرسش‌نامه محقق ساخته بود. اعتبار ابزارتحقق (پرسش‌نامه) با استفاده از نظرات صاحب‌نظران حوزه تربیت بدنی و تعیین پایایی آن از روش آزمون–بازآزمون انجام شد، در روش آزمون-بازآزمون تعداد 30 پرسش‌نامه در دو نوبت بین 30 نفراز سالمندان توزیع شد و ضریب آلفای کرونباخ پرسش‌نامه 0/80 مشخص گردید. یافته‌های تحقیق از طریق برنامه SPSS نسخه 13 مورد تجزیه و تحلیل قرار گرفت. برای توصیف یافته‌ها از آمار توصیفی و برای تعیین رابطه بین بعضی از متغیرها از آزمون‌های همبستگی و مجذور کای استفاده شد. یافته‌ها: پارک‌ها اولین اولویت برای گذران فراغت سالمندان می باشد،حدود 59/7درصد از اوقات فراغتشان در حد متوسط رضایت دارند و 74درصد ورزش می کنند، بیشترین فعالیت ورزشی آن‌ها پیاده‌روی است و 48/2درصد ازدستگاه‌های بدن‌سازی شهرداری استفاده نمی‌کردند و 41/7درصد استفاده از دستگاه‌های بدن‌سازی را برای سالمندان در حد زیاد و خیلی زیاد مناسب می‌دانستند. هم چنین بین سن و میزان اوقات فراغت و بین جنسیت و میزان رضایت از دستگاه‌های بدن‌سازی شهرداری رابطه معنی‌دار وجود دارد(P≤0/01). اگر چه بین میزان رضایت از دستگاه‌های بدن‌سازی و سن ارتباط معنی‌دار دیده نشد (P≤0/05). نتیجه‌گیری: سالمندان به‌طور متوسط از اوقات فراغت خود رضایت دارند و انجام فعالیت‌های بدنی نقش مهمی در میزان رضایت از گذران اوقات فراغت آن‌هادارد و وجود امکانات ورزشی مناسب در پارک‌ها باعث تشویق سالمندان برای انجام حرکات ورزشی خواهد شد. بنابراین توسعه این امکانات با توجه به نیازهای فراغتی و بدنی سالمندان توصیه می‌شود.

Title: تأثیر یک برنامه مراقبت از سالمند بر نگرش دانشجویان پرستاری نسبت به سالمند

http://salmandj.uswr.ac.ir/article-1-662-fa.html

اهداف: مراقبت از سالمند، بخش مهمی از وظایف حرفه‌ای پرستار است. در برخی از مطالعات گزارش شده است که دانشجویان پرستاری نسبت به سالمندان و مراقبت از آن‌ها نگرش منفی داشته و تمایلی برای انتخاب محیط‌های مراقبت از سالمندان در آینده شغلی خود ندارند. همچنین اعتقاد بر این است که تجارب بالینی روی نگرش به سالمندان اثر می‌گذارد. لذا پژوهش حاضر با هدف بررسی تأثیر مراقبت از سالمند بر نگرش دانشجویان پرستاری دانشگاه علوم پزشکی کاشان نسبت به سالمندان انجام شد. مواد و روش‌ها: این پژوهش از نوع مداخله‌ای است که در آن تعداد 59 دانشجوی سال چهارم پرستاری مورد پژوهش قرار گرفتند. نمونه گیری با روش آسان انجام شد. دانشجویان ترم 8 (32 نفر) در گروه کنترل و دانشجویان ترم 7 (27 نفر) در گروه مداخله قرار گرفتند. ابتدا از هر دو گروه پیش آزمون گرفته شد، سپس در گروه مداخله، یک برنامه 10 روزه مراقبت از سالمند اجرا شد. یک روز پس از روز دهم، پس آزمون از هر دو گروه انجام شد. جهت بررسی نگرش دانشجویان از پرسشنامه KAOP استفاده شد. داده‌ها با استفاده از آزمون t مستقل، t زوج، ANOVA  ،chi-square و آزمون دقیق فیشر در نرم‌افزار SPSS 11/5 تجزیه و تحلیل شد. یافته‌ها: در پژوهش حاضر، %40/7 از دانشجویان مرد و %59/3 زن بودند. میانگین نمرات نگرش دانشجویان در دو گروه کنترل و مداخله در ابتدای مطالعه تفاوت معنی‌داری نداشت (0/62=P). میانگین نمرات نگرش در گروه مداخله در ابتدای مطالعه 118/18 و در انتهای مطالعه 119/62 بود (0/62=P). همچنین میانگین نمرات نگرش در گروه کنترل در ابتدای مطالعه 119/4 و در انتهای مطالعه 125/37 بود (0/01=P). نتیجه‌گیری: برنامه مراقبت 10 روزه از سالمندان، تاثیر محسوس و معنی‌داری بر نگرش دانشجویان پرستاری به سالمندی نداشت، درحالی‌که گردش یک روزه در سرای سالمندان با بهبود نگرش به سالمندی همراه بود. احتمالا شرایط نگهداری سالمندان بر چگونگی تغییر نگرش تاثیر داشته است. مطالعه بیشتر در این زمینه پیشنهاد می‌شود.

Title: تأثیر تغییرات وزن اندام بر ثبت الکترومیوگرافی زمان واکنش ساده و افتراقی مردان مسن

http://salmandj.uswr.ac.ir/article-1-680-fa.html

اهداف: اثر تغییر نیروی انقباضی یکی از عوامل مهم سازماندهی و اجرای تکالیف زمان واکنش است که غالب افراد مسن با افزایش وزن خود با این متغیر درگیر هستند. هدف از تحقیق حاضر بررسی اثر تغییرات وزن اندام بر ثبت الکترومیوگرافی زمان واکنش ساده و افتراقی حرکت فلکشن آرنج مردان مسن است. مواد و روش‌ها: از آزمودنی‌ها (16 نفر با میانگین سنی 59/18 سال) خواسته شد تا در پاسخ به محرک های شنیداری (ساده و افتراقی)، حرکت فلکشن ساعد به سمت نقطه هدف در فاصله 60سانتیمتری را انجام دهند. در 50درصد از کوشش‌های انجام گرفته وزن اندام درگیر این حرکت به مقدار 1/2 کیلوگرم افزایش می‌یافت و در همه کوشش‌های انجام گرفته تغییرات زمان واکنش و تنش عضلانی توسط دستگاه الکترومیوگرام ثبت می‌گردید. یافته‌ها: نتایج آزمون تحلیل واریانس عاملی با اندازه‌گیری‌های تکراری نشان داد که اثرات اصلی هر دو فاکتور افزایش وزن اندام (0/001=P) و تعداد محرک (0/001=P) بر روی بخش PMT معنادار بوده است. در حالی که اثر افزایش محرک از حالت ساده به افتراقی بر بخش MT‌ معنادار نبوده است (0/189=P). هیچ تعاملی بین فاکتور افزایش وزن اندام و تعداد محرک بر روی بخش‌های PMT (P=0/888) و MT (P=0/91) وجود نداشت. نتیجه‌گیری: این یافته‌ها بیان می‌کنند، عواملی که موجب تغییر در اینرسی اندام و به‌طورکلی عوامل حرکتی می‌شوند، با درگیرکردن هم‌زمان بخش حرکتی و پیش‌حرکتی زمان واکنش می‌توانند عملکرد افراد مسن را به شدت تحت تاثیر قرار دهند.

Title: تعیین روایی و پایایی پرسش‌نامه سطح فعالیت فیزیکی (CHAMPS) در سالمندان تهرانی

http://salmandj.uswr.ac.ir/article-1-778-fa.html

اهداف: تهیه نسخه فارسی و تعیین روایی و پایایی پرسشنامه سطح فعالیت فیزیکی (CHAMPS) در سالمندان تهرانی اهداف این مطالعه بودند. مواد و روش‌ها: این مطالعه به صورت توصیفی – تحلیلی مقطعی انجام گردید، 200 سالمند تهرانی وارد مطالعه شدند و پرسشنامه های سطح فعالیت فیزیکی چامپس، SF-36 و سطح ارتقاء دهنده سلامت والکر، توسط آنها تکمیل گردید و همچنین شاخص توده جسمانی و آزمون شش دقیقه راه رفتن در آنها ارزیابی شد. فرآیند ترجمه و معادل سازی بر اساس پروتکل پروژه بین المللی ابزار کیفیت زندگی (IQOLA) انجام شد. پایایی در دفعات آزمون پس از 2 هفته توسط 30 نفر سالمند و همچنین توسط ضریب آلفای کرونباخ محاسبه شد. یافته‌ها: بیش از 85درصد آیتم ها کیفیت ترجمه مطلوبی داشتند. مطلوبیت ترجمه تمام آیتم ها با استفاده از پیشنهادات مترجمین به دست آمد. روایی ظاهری مناسب نسخه فارسی پرسشنامه سطح فعالیت فیزیکی چامپس حاصل شد. بیش از 96درصد سؤالات همبستگی بالایی را با خرده مقیاس مربوطه نشان دادند. ضریب همبستگی درون رده ای (ICC) برای نمره خرده مقیاس ها و نمره کل بیش از 0/49 به دست آمد. همچنین در مورد هم خوانی درونی ضریب آلفای کرونباخ پرسشنامه 0/76 و ضریب همبستگی آزمون- آزمون مجدد در دامنه 0/64-0/78 درصد بدست آمد. ضریب روایی محتوی (CVI) برابر با 0/83 بود. برای روایی همگرا، ضریب همبستگی بین نمرات پرسش‌نامه چامپس با آزمون شش دقیقه راه رفتن و شاخص توده جسمانی به ترتیب 0/25 و 0/19 به‌دست آمد. برای روایی همزمان، ضریب همبستگی بین نمرات پرسش‌نامه چامپس با کیفیت زندگی و سطح ارتقادهنده سلامت به ترتیب 0/33 و 0/42 به‌دست آمد. نتیجه‌گیری: فرآیند ترجمه و معادل سازی پرسش‌نامه سطح فعالیت فیزیکی چامپس از کیفیت مطلوب و قابل قبولی برخوردار است. همچنین نسخه فارسی این پرسش‌نامه از روایی ظاهری، محتوی، همگرا و همزمان، و پایایی در دفعات آزمون (نسبی و مطلق) و هم‌خوانی درونی در حد قابل قبولی در جامعه مورد نظر برخوردار است. بنابراین نسخه فارسی این پرسش‌نامه را می توان در تحقیقات به عنوان ابزار بررسی سطح فعالیت فیزیکی در سالمندان به کار برد.

Title: تأثیر یک جلسه فعالیت مقاومتی بر میزان BDNF، IGF-1 و IGFBP-3 در افراد سالمند

http://salmandj.uswr.ac.ir/article-1-635-fa.html

اهداف: هدف از انجام این پژوهش بررسی تأثیر یک جلسه فعالیت مقاومتی بر میزان عامل نوروتروفیک مغزی (BDNF)، عامل رشد شبه انسولین-1 (IGF-1) و پروتئین متصل به عامل رشد شبه انسولین-3 (IGFBP-3) در افراد سالمند بود. مواد و روش‌ها: در این مطالعه 22 فرد سالمند سالم (سن 4/23±67 سال و 0/3±24/5=BMI) شرکت داشتند. 72 ساعت پس از تعیین حداکثر قدرت بیشینه (با استفاده از آزمون 1-RM)، آزمودنی‌ها دریک جلسه فعالیت مقاومتی با شدت 75 درصد 1-RM شرکت کردند. در این پژوهش 3 نمونه خونی (هر وهله 10 میلی لیتر) در قبل، بلافاصله و 30 دقیقه بعد از فعالیت مقاومتی از ورید بازویی آزمودنی‌ها گرفته شد. به‌منظور تعیین میزان BDNF، IGF-1 و IGFBP-3 سرم، از روش الایزا استفاده شد. جهت تعیین تغییرات درون گروهی سه فاکتور مذکور در پاسخ به فعالیت مقاومتی، از روش آماری Repeated Measures (1×3) استفاده شد. سطح معنی‏‌داری P≤0/05 در نظر گرفته شد. یافته‌ها: نتایج این پژوهش نشان داد که بلافاصله پس از فعالیت مقاومتی، افزایش معنی‏داری در میزان BDNF سرم رخ می‌دهد، اما 30 دقیقه پس از اجرای پروتکل، نسبت به قبل از فعالیت تغییر معنی‏داری مشاهده نشد (P≤0/05). همچنین نشان داده شد که در میزان IGF-1، بلافاصله پس از یک جلسه فعالیت مقاومتی افزایش معنی‏داری وجود دارد، اما 30 دقیقه پس از اجرای پروتکل، نسبت به قبل از فعالیت تغییر معنی‏داری مشاهده نشد (P≤0/05). در نهایت نیز تغییر معنی‏داری در میزان IGFBP-3 در بلافاصله و 30 دقیقه پس از اجرای پروتکل مقاومتی، نسبت به قبل از فعالیت، مشاهده نشد (P≤0/05). نتیجه‌گیری: به طور کلی نتایج این پژوهش نشان داد، در پاسخ به فعالیت مقاومتی میزان BDNF و IGF-1 در بلافاصله پس از فعالیت، افزایش معنی‌‏داری می‌یابند، اما تغییر معنی‏‌داری در میزان IGFBP-3، مشاهده نشد. بنا بر نتایج این پژوهش، به نظر می‌رسد، فعالیت مقاومتی باعث ایجاد تغییراتی مثبت، در میزان فاکتورهای نوروتروفیکی درگیر در فرآیند حافظه و یادگیری می‌گردد. این امر در نهایت ممکن است موجب کاهش میزان شیوع بیماری‎های عصبی مرتبط با اختلال حافظه و یادگیری همچون آلزایمر، افسردگی و دی منتیا در افراد سالمند گردد.

Title: کاربرد روش MI-GEE در مقایسه دو روش درمانی برای زنان بیشتر از 50 سال مبتلا به استئوآرتریت شدید زانو

http://salmandj.uswr.ac.ir/article-1-701-fa.html

اهداف: استئوآرتریت شدید زانو در زمره یکی از 5 بیماری قرار دارد که بیشترین سهم را در ایجاد ناتوانی در زنان و مردان غیربستری سالمند دارد. در این پ‍ژوهش دو روش درمانی در زنان گروه سنی 75-50 سال مورد مقایسه قرار گرفتند. مواد و روش‌ها: در این پژوهش تعداد 40 بیمار زن در گروه سنی 75-50 سال که به استئوآرتریت شدید زانو مبتلا بودند، مورد مطالعه قرار گرفتند. پس از تصادفی سازی در دو گروه با تعداد یکسان، روش درمان معمول برای گروه اول (کنترل) و روش درمان معمول همراه با کشش مفصلی برای گروه دوم (مورد) درنظرگرفته شدند. متغیر پاسخ در این مطالعه، شدت درد بیماران بود که به صورت دو حالتی (شدید، ضعیف) و در 10 جلسه درمانی مورد سنجش قرار گرفت. به علت وجود داده‌های گم شده، داده‌ها به روش رگرسیونی multiple-imputation-based generalized estimating equations (MI-GEE) و به کمک نرم‌افزار R مورد تجزیه‌و‌تحلیل قرار گرفتند. نتایج به صورت نسبت‌های شانس و فواصل اطمینان مربوطه گزارش شده‌اند. یافته‌ها: در مدل رگرسیونی چند متغیره، ارتباط معنی‌دار آماری بین روش درمان و شدت درد به‌دست آمده است. نسبت شانس درد در روش درمان معمول همراه با اعمال کشش مفصلی نسبت به روش درمان معمول برابر 0/28 می‌باشد (0/65-0/12 :CI:95%). نتیجه‌گیری: درمان معمول همراه با اعمال کشش مفصلی در مقایسه با درمان معمول سبب کاهش معنی‌دار درد بیماران مبتلا به استئوآرتریت شدید زانو می‌گردد.

Title: تئوری‌های بیوشیمیایی و ژنتیکی فرایند پیری

http://salmandj.uswr.ac.ir/article-1-450-fa.html

پیری، نتیجۀ تجمع پیشروندۀ تغییرات مختلف در بدن می‌باشد که با گذشت زمان، با کاهش تدریجی کارایی بسیاری از اعمال فیزیولوژیک نرمال و ظرفیت حفظ هموستاز، همراه بوده و باعث افزایش احتمال بیماری و مرگ افراد می‌گردد. محققان، برای درک فرایندهای پیری، افزایش طول عمر و بهبود کیفیت زندگی‌، آزمایشات مختلف به‌ویژه بر روی مدل‌های حیوانی انجام داده و بر اساس آن‌ها، بیش از 300 تئوری مرتبط با پیری را مطرح نموده‌‌اند که تا حدی با هم همپوشانی دارند. در این مقاله، سعی شده است که به بعضی از مهم‌ترین این تئوری‌ها و با تمرکز بیشتر به تئوری اپی‌ژنتیک پیری پرداخته شود. دو تا از شاخص‌ترین تئوری‌های بیوشیمیایی پیری، تئوری‌های رادیکال آزاد و میتوکندری هستند. با توجه به اینکه پیری نرمال، نتیجۀ تعادل بین آسیب و ترمیم است، در این تئوری‌ها، شناخت استرس اکسیداتیو مرتبط با آسیب حاصل از گونه‌های فعال اکسیژن (ROS)، مسیر سیگنالینگ پاسخ استرس و آنزیم‌های آنتی‌اکسیدانتی، حائز اهمیت می‌باشند. بر اساس تئوری‌های ژنتیکی پیری، رخداد پیری، حاصل برنامۀ ژنتیکی کنترل شدۀ‌ تکامل و بلوغ است. ساعت میتوتیک پیری هم که بوسیلۀ تلومرها، عناصر ژنتیکی ضروری پایدارکنندۀ انتهای کروموزومی، راهنمایی می‌شود، قابل توجه می‌باشد. طول تلومرها که بعد از هر تقسیم سلولی کوتاه می‌شود، می‌تواند توسط موادغذایی مختلف و شرایط اپی‌ژنتیک تلومری تنظیم شود. علاوه بر این، روند پیری، نه فقط بوسیلۀ ژن‌ها و موتاسیون، بلکه همچنین بواسطۀ تأثیرات محیطی و اپی‌ژنتیکی به‌خصوص در نیمۀ دوم زندگی، تحت تأثیر قرار می‌گیرد. تغییرات اپی‌ژنتیکی، شامل تغییرات در بیان ژن بدون تغییر در ترادف DNA می‌باشند. اهمیت عملکردی و بیولوژیکی تغییرات اپی‌ژنتیکی در طی فرایند پیری، با فقدان گلوبال متیلاسیون DNA، تغییرات هیستونی و هیپرمتیلاسیون پروموتور ژن‌ها، همراه می‌باشد. جهت دستیابی به رویای بشر به زندگی طولانی‌تر و سالم‌تر، دستکاری رژیم غذایی با استفاده از مکمل‌ها و آنتی‌اکسیدانت‌ها، درک نقش کلیدی محدودیت کالری در افزایش طول عمر بواسطۀ فعالیت پروتئین Sirtuin، تمرینات ورزشی و شناخت عوامل محیطی تاًثیرگذار بر تغییرات اپی‌ژنتیکی در فرایند پیری، از ضروری‌ترین موضوعات تحقیقاتی در قرن 21، می‌باشد.
